# Exosomes Are Unlikely Involved in Intercellular Nef Transfer

**DOI:** 10.1371/journal.pone.0124436

**Published:** 2015-04-28

**Authors:** Xiaoyu Luo, Yan Fan, In-Woo Park, Johnny J. He

**Affiliations:** Department of Cell Biology and Immunology, Graduate School of Biomedical Sciences, University of North Texas Health Science Center, Fort Worth, Texas, 76107, United States of America; Helmholtz Zentrum Muenchen—German Research Center for Environmental Health, GERMANY

## Abstract

HIV-1 Nef is an important pathogenic factor for HIV/AIDS pathogenesis. Several recent studies including ours have demonstrated that Nef can be transferred to neighboring cells and alters the function of these cells. However, how the intercellular Nef transfer occurs is in dispute. In the current study, we attempted to address this important issue using several complementary strategies, a panel of exosomal markers, and human CD4+ T lymphocyte cell line Jurkat and a commonly used cell line 293T. First, we showed that Nef was transferred from Nef-expressing or HIV-infected Jurkat to naïve Jurkat and other non-Jurkat cells and that the transfer required the membrane targeting function of Nef and was cell density-dependent. Then, we showed that Nef transfer was cell-cell contact-dependent, as exposure to culture supernatants or exosomes from HIV-infected Jurkat or Nef-expressing Jurkat and 293T led to little Nef detection in the target cells Jurkat. Thirdly, we demonstrated that Nef was only detected to be associated with HIV virions but not with acetylcholinesterase (AChE+) exosomes from HIV-infected Jurkat and not in the exosomes from Nef-expressing Jurkat. In comparison, when it was over-expressed in 293T, Nef was detected in detergent-insoluble AChE+/CD81^low^/TSG101^low^ exosomes, but not in detergent-soluble AChE-/CD81^high^/TSG101^high^ exosomes. Lastly, microscopic imaging showed no significant Nef detection in exosomal vesicle-like structures in and out 293T. Taken together, these results show that exosomes are unlikely involved in intercellular Nef transfer. In addition, this study reveals existence of two types of exosomes: AChE+/CD81^low^/TSG101^low^ exosomes and AChE-/CD81^high^/TSG101^high^ exosomes.

## Introduction

Intercellular protein transfer has been recognized as a common phenomenon for cell-cell communication in multi-cellular organisms including plants and animals; it can occur among immune cells and nonimmune cells [1, 2]. The underlying mechanisms can be cell-cell contact-dependent or independent [3–5]. The cell-cell contact-dependent protein transfer includes tunneling nanotubes (TNT) and trogocytosis. TNT are characterized by long cytoplasmic bridges that enable long-range cell-cell communication and function to transfer large cellular structures such as vesicles and organelles [6, 7], while trogocytosis involves formation of close intercellular structure such as synapse and transfer of plasma membrane fragments from one cell to the other leading to molecular reshuffling between adjacent cells, particularly immune cells [4, 8]. In contrast, cell-cell contact-independent protein transfer is accomplished through release of protein-bearing membrane vesicles (MV) or exosomes by one cell and uptake of protein-bearing MV or exosomes by the other cell [5, 9]. Intercellular protein transfer regulates immune response and other cellular function of neighboring cells such as cellular homoeostasis and anti-tumor activities [10–13].

As a retrovirus, HIV-1 genome encodes three structural proteins Gag, Pol and Env and six accessory proteins Tat, Rev, Nef, Vpr, Vpu and Vif [14–16]. All six accessory proteins are important for various aspects of HIV-1 replication and pathogenesis [17, 18]. *In vivo* studies have shown that Nef is indispensable for HIV-1 pathogenesis. Expression of *nef* in mice leads to an AIDS-like disease [19]; while *nef* deletion or defect is linked to lower viral load and attenuated diseases in humanized mice, non-human primates and humans [20–27]. Nef is about 27 kDa and myristoylated at the second amino acid glycine; the myristoylation targets Nef onto the plasma membrane [28, 29], although it is also detected in cytosol [30]. In addition, Nef is detected in HIV virion particles [31]. Nef localization on the plasma membrane confers Nef several important functions such as protein trafficking, down-regulation of cell surface receptors, alteration of intracellular signaling, and enhancement of HIV-1 infectivity [28, 32–39].

Several studies have recently uncovered that Nef is transferred among cells, suggesting that intercellular Nef transfer could contribute to HIV disease progression such as *in vivo* CD4+ T cell depletion. Intercellular HIV-1 Nef transfer has been noted between HIV-infected macrophages and B cells [40] and between HIV-infected/Nef-expressing CD4+ T lymphocytes and uninfected CD4+ T cells [41, 42]. We have recently reported intercellular HIV-1 Nef transfer between HIV-infected/Nef expressing CD4 T lymphocytes and hepatocytes [43]. Both cell-cell contact-independent mechanisms such as tunneling nanotubes and cell-cell contact-independent mechanisms such as exosomes and other extracellular vesicles have been proposed for intercellular Nef transfer [40–42, 44–46]. Thus, elucidation of the exact mechanisms of intercellular Nef transfer is warranted for further addressing the critical roles of HIV-1 Nef in HIV-1 pathogenesis. In the current study we wished to define the underlying mechanisms of intercellular Nef transfer using a combined cell biology, virology, biochemistry and microscopic imaging approach.

## Materials and Methods

### Cells culture and reagents

Human embryonic kidney cell line 293T and human T lymphoblastoid cell line Jurkat E6-1 were obtained from American Tissue Culture Collection (ATCC, Manassas, VA) and maintained in Dulbecco’s modified Eagle’s medium (DMEM, Lonza, Walkersville, MD) or Roswell Park Memorial Institute 1640 medium (RPMI-1640, Lonza), respectively. Both media were supplemented with 10% Fetal bovine serum (Hyclone, Logan, UT) and 1% Penicillin-streptomycin-glutamine (Lonza) at 37°C with 5% CO_2_. Exosome-free medium used in all the studies was obtained by ultracentrifugation of the complete (supplemented with serum and antibiotic) culture medium at 100,000 *g* for 16 hr (SW28 rotor, Beckman counter), verified by the AChE activity assay (see below). Mouse anti-Nef antibody (sc-65904), rabbit anti-Myc antibody (sc-789), and mouse anti-Cytochrom C antibody (sc-13561) were purchased from Santa Cruz Biotechnology (Santa Cruz, CA). Phycoerythrin (PE)-conjugated mouse-anti-p24 antibody (KC57) was purchased from Beckman Counter (Brea, CA). Mouse anti-p24 antibody derived from p24 hybridoma cells (#1513), rabbit anti-Nef antibody (#2949), and mouse anti-Nef (#1539) were obtained from NIH AIDS Reagent Program, and donated by Dr. Bruce Chesebro of National Institute of Allergy and Infectious Diseases, Hamilton, Montana [47], Dr. Ronald Swanstrom of University of North Carolina at Chapel Hill [48], and Dr. K. Krohn and Dr. V. Ovod of University of Tampere, Institute of Biochemical Sciences, Finland [49], respectively. Rabbit anti-GFAP antibody (Z0334) was purchased from Dako (Carpinteria, CA). Rabbit anti-GFP antibody (# 632592) was purchased from Clontech (Mountain View, CA). Mouse anti-CD81 antibody (# 555675) was purchased from BD PharMingen (San Diego, CA). Rabbit anti-CD9 antibody (EXOAB-CD9A-1) and rabbit anti-HSP70 antibody (EXOAB-Hsp70A-1) were purchased from System Biosciences (Mountain View, CA). Rabbit anti-TSG101 antibody (T5701), OptiPrep (60% iodixanol w/v in water), acetylthiocholine, and 5,5'-dithiobis-(2-nitrobenzoic acid) were purchased from Sigma-Aldrich (St. Louis, MO). Sheep anti-mouse IgG-HRP and donkey anti-rabbit IgG-HRP were purchased from GE Healthcare (Little Chalfont, Buckinghamshire, UK). Goat-anti-mouse Alexa-Fluor-555 antibody and goat-anti-rabbit Alexa-Fluor-488 was purchased from Molecular Probes (Eugene, Oregon, USA). Enhanced chemiluminesence (ECL) reagents for Western blot detection were made in house and the protease inhibitor cocktail were purchased from Roche (Indianapolis, IN).

### Plasmids

pNef.myc and pNef.GFP were constructed as previously described [50]. pCD81.GFP was constructed in the context of the pEGFP-N3 backbone (Clontech) using pCDNA3.CD81 [51, 52] as respective templates with primers: 5'-GA CTG GGA TCC GTA CAC GGA GCT GTT CCG GAT GCC-3’ and T7. NL4-3ΔNef was constructed by first cloning an Xho I/Nae I NL4-3 Nef fragment into the pBlueScript KS+ vector (Stratagene, La Jolla, CA), followed by site-directed mutagenesis using a Stratagene mutagenesis kit and primers: 5’-TCT CGA GAC CTA TGA AAA CAT GGA GCA ATC ACA AG -3’ and 5’-CT TGT GAT TGC TCC ATG TTT TCA TAG GTC TCG AGA-3’ and cloning the mutated Xho I/Nae I fragment to replace the corresponding fragment of the NL4-3 backbone. HIV-1 reporter virus gagi and NLGi plasmids were generously provided by Dr. B. K. Chen of Mount Sinai School of Medicine [53, 54]. NLGi ΔNef was constructed by Xho I digestion, followed by filled-in with T4 polymerase. HXB2 Eli and HXB2 Eli 2GA were described previously [55].

### Transfections and Western blotting

293T were transfected using the standard calcium phosphate precipitation method, which usually gave rise to more than 90% transfection efficiency. Cells or exosomes were lysed by RIPA buffer (10 mM Tris.Cl, pH 8.0, 1 mM EDTA, 0.5 mM EGTA, 1% Triton X-100, 0.1% sodium deoxycholate, 0.1% SDS, 140 mM NaCl, supplemented with protease inhibitor and phenylmethylsulfonyl fluoride (Sigma). Lysates were sonicated and cleared of cell debris by centrifugation, suspended in 1X SDS sample buffer, incubated at 37°C for 30 min or 100°C 10 min, and separated on a 10% SDS-PAGE. Proteins were then transferred onto nitrocellulose membrane and detected for GFP expression at a wavelength of 488 nm (for GFP or GFP fusion proteins), or probed with desired primary and secondary antibodies and the enhanced chemiluminescence reagents.

### Virus production and infection

293T were transfected with pNL4-3 plasmid, the culture medium was collected and spun through a 20% sucrose cushion at 100,000 *g* for 2 hr to obtain HIV of high purity. Purified virus was suspended in PBS, aliquoted and stored in liquid nitrogen. Viral titers were then determined by the reverse transcriptase assay. For infection, Jurkat (1 x 10^6^) were incubated with NL4-3 equivalent to 10,000 cpm RT in 1 ml complete RPMI at 37°C and 5% CO_2_ for 4 hr. The cells were then washed with PBS twice to remove the input virus and continued to culture in fresh medium at a density of 0.3–1 x 10^6^ cell/ml. The percentages of infected cells were determined by p24 staining and flow cytometry.

### Reverse transcriptase (RT) assay

Viruses were pelleted and suspended in 10 μl dissociation buffer (50 mM Tris.HCl, pH 7.5, 0.25% Triton X-100, 20% glycerol, 1 mM DTT, and 0.25 M KCl), followed by three rounds of frozen and thawing. The virus lysates were added 35 μl RT assay buffer (50 mM Tris.HCl, pH 7.5, 1 mM DTT, 10 mM MgCl2, and 0.25% Triton X-100), 5 μl 1 mg/ml poly (A).(dT)15 (Roche, Indianapolis, IN) and 1 μl [^3^H]-thymidine 5′-triphosphate tetrasodiun salt (ICN, Irvine, CA) and incubated at 37°C for 1 hr. The mixture was spotted onto a DE81 ion exchange chomatographic disk (Whatman, Clifton, NJ). The disks were washed with 2X SSC (0.3 M NaCl and 30 mM Na citrate, pH 7.0) three times, 5 min each, dehydrated with 100% ethanol, air dried and then determined for ^3^H incorporation using a scintillation counter (Perkin Elmer, Waltham, MA). The RT activity was expressed as counts per minute (cpm).

### Exosome isolation

Transfected 293T, GFP/Nef.GFP-expressing Jurkat or HIV-infected Jurkat (with an infection efficiency of 70%) were cultured in the exosome-free medium for 3 days. At the end of the culture, the culture medium was collected and processed as stated below. The first step was to remove cells and cell debris, consisting of three sequential centrifugations: 300 *g* for 10 min, 2,000 *g* for 10 min (alternatively, filtration through 0.22 μm filter), and 10,000 *g* for 30 min. Between each centrifugation step, the supernatant was carefully recovered and used for the next centrifugation. The next step was to obtain crude exosomes by subjecting the cleared supernatant from the first step to ultracentrifugation at 100,000 *g* for 70 min (SW28 rotor, Beckman, Indianapolis, IN). Following the ultracentrifugation, the supernatant was carefully removed and discarded, while the pellet was saved and either lysed in the RIPA buffer for Western blotting, or suspended in PBS for the next step of OptiPrep gradient purification, or exosome-free medium for exosome uptake analysis. The third step is to further fractionate the crude exosomes through a 6–18% OptiPrep gradient. Specifically, the crude exosomes in PBS (about 500 μl) from the second step above were loaded on the top of 5 ml 6–18% OptiPrep gradient that was prepared using a gradient maker Hoefer SG15 (Hoefer, Inc., Hilliston, MA), followed by ultracentrifugation at 250,000 *g* for 1.5 hr (SW55Ti rotor, Beckman). OptiPrep was diluted in 235 mM KCl, 12 mM MgCl_2_, 25 mM CaCl_2_, 30 mM EGTA, 150 mM Hepes-NaOH, pH 7.0. A total of 12 fractions from top to bottom, 450 μl each was collected. Trichloroacetic acid (TCA) precipitation was used to recover the proteins from each fraction. Briefly, TCA was added to each fraction with a final concentration of 20%, the mixture was incubated on ice for 15 min and then spun to obtain the precipitates. The precipitates were washed with cold acetone twice, dried, and dissolved in 1X SDS loading buffer for Western blotting. Alternatively, the fractions were diluted 4 ml PBS and spun at 100,000 *g* for 70 min to obtain exosome pellets. The pellets were lysed in the RIPA buffer for Western blotting.

### Acetylcholinesterase activity assay

Exosome preparation, e.g., fractions obtained from the OptiPrep gradient centrifugation (15 μl) was mixed with 85 μl 1.25 mM acetylthiocholine and 100 μl 0.1 mM 5,5'-dithiobis-(2-nitrobenzoic acid) in a 96-well plate. The mixture was incubated at room temperature until a yellowish color was developed. Then, the optical density (OD) at a wavelength of 450 nm was determined using a 96-well plate reader (Bio-Rad, Hercules, CA).

### Immunofluorescence staining and microscopic imaging

293T were transfected with indicated plasmids and re-plated in a 24-well plate containing polylysine-coated coverslips 16 hr post transfection. The cells were fixed with 4% paraformaldehyde and either mounted directly for imaging under a Zeiss Axiovert 200 microscope (Carl Zeiss, Thornwood, NY), or permeabilized in 0.1% Triton, blocked with PBS-BB (1% non-fat milk, 0.2% bovine serum albumin 0.3% Triton), stained with appropriate primary and secondary antibodies, followed by imaging under a Zeiss Axiovert 200 microscope. For exosome uptake experiments, 293T were cultured in a polylysine-coated glass bottom dish for live cell imaging (In Vitro Scientific, Sunnyvale, CA) and incubated with exosome preparations. Images of live cells were taken using a Zeiss Axiovert 200 microscope.

### Data analysis

All values were expressed as mean ± SD of triplicate samples. All comparisons were made using two-tailed Student’s *t*-test. A *p* of < 0.05 was considered statistically significant (*), *p* < 0.01 highly significant (**). All data were representative of multiple repeated experiments.

## Results

### Intercellular Nef transfer from Nef-expressing cells to Jurkat, THP-1 and human primary astrocytes

**Fig 1:**
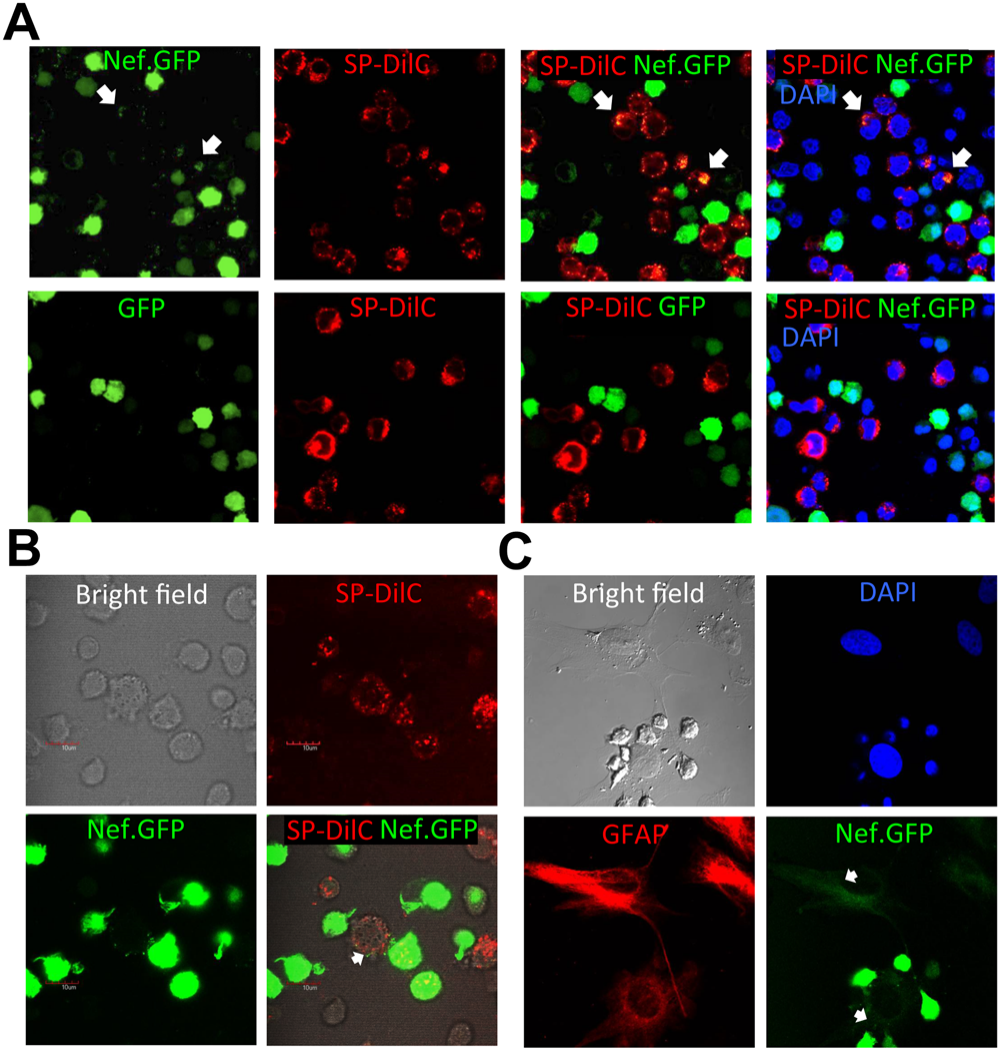
Intercellular HIV-1 Nef transfer. Nef.GFP-expressing Jurkat (0.5 x 10^6^) were co-cultured with 0.5 x 10^6^ of SP-DilC-labeled Jurkat (**A,** top panels), SP-DilC-labeled THP-1 (**B**), or HPA (**C**) in a volume of 200 μl medium in a 24-well plate (i.e., at a cell density of 0.5 x 10^6^/ml) for 16 hr. GFP-expressing Jurkat were used as a control (**A,** bottom panels). HPA were identified by GFAP staining, and DAPI staining was also performed to discern HPA from Nef.GFP-expressing Jurkat by the size of the nuclei (**C**). Nef transfer from Nef.GFP-expressing Jurkat to Jurkat (**A**), THP-1 (**B**) and HPA (**C**) was shown by arrows.

Previous studies have shown intercellular Nef transfer between macrophage and B cells, between CD4+ T cells and CD4+ T cells, and between CD4+ T cells and hepatocytes [40–43]. To determine whether intercellular Nef transfer could occur between Nef-expressing CD4+ T lymphocytes and other types of cells, we took advantage of a Jurkat cell clone stably expressing HIV-1 Nef.GFP fusion protein [56] and used it as the donor cells in the co-culture assay with target cells Jurkat, human monocytic cells THP-1, or human primary astrocytes (HPA). The target cells were either pre-labeled with cell-permeable labeling dye SP-DilC (for Jurkat and THP-1) or stained for glial fibrillary acidic protein (GFAP), an astrocyte marker for HPA. Consistent with previous findings [41, 42], co-culture between Nef.GFP-expressing Jurkat and regular Jurkat led to Nef.GFP detection in the regular Jurkat (Fig 1A, top panels). Similarly, co-culture with Nef.GFP-expressing Jurkat led to Nef detection in THP-1 (Fig 1B) and HPA (Fig 1C). There was no GFP detection in regular Jurkat when GFP-expressing Jurkat were used as the donor cells (Fig 1A, bottom panels) and in THP-1 (data not shown) and HPA (S1 Fig).

### Intercellular Nef transfer in the context of HIV infection

**Fig 2:**
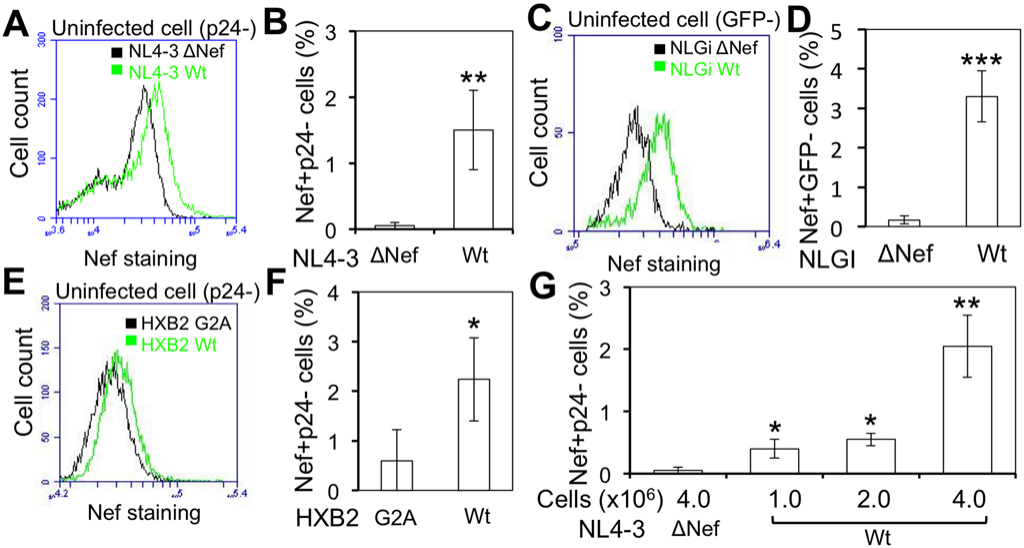
HIV-1 Nef transfer from HIV-infected cells to bystander cells. Jurkat were infected with HIV-1 NL4-3 (Wt) or *nef*-deleted NL4-3 (ΔNef) (**A & B**), HXB2 (Wt) or Nef mutated HXB2 (G2A) (**E&F**). When the infection reached 50%, determined by p24 staining, the cells were double stained using a rabbit anti-HIV-1 Nef antibody with Alexa anti-rabbit 488 and PE-conjugated mouse anti-p24 antibody. The cells were analyzed by flow cytometry for Nef staining fluorescence intensity in p24- cells (**A & E**), and the percentage of Nef+p24- cells (**B & F**). MT4 were infected with NLGi (Wt) or *nef*-deleted NLGi (ΔNef) (**C & D**). Co-culture of infected MT4 with Jurkat at 1:1 ratio was performed when MT4 infection reached 80% determined by GFP expression. The cells were collected 48 h post co-culture and stained using a mouse anti-Nef antibody (1539) with Alexa anti-mouse 647. The cells were analyzed by flow cytometry for Nef staining fluorescence intensity in GFP- cells (**C**), and the percentage of Nef+GFP- cells (**D**). The data were mean ± SD of triplicates and representative of three independent experiments. *, *p* < 0.05 **, *p* < 0.01. **G.** Jurkat were infected with HIV-1 NL4-3 Wt or ΔNef. When the infection efficiency reached more than 90%, the cells were co-cultured with Jurkat at a ratio of 1:1 but at a cell density of 1 x 10^6^, 2 x 10^6^, or 4 x 10^6^ cells /ml for 16 hr. At the end of the co-culture, the cells were double stained using rabbit anti-HIV-1 Nef antibody with Alexa anti-rabbit 488 and PE-conjugated mouse anti-p24 antibody. The cells were analyzed by flow cytometry for Nef+p24- cells. The data were mean ± SD of triplicates and representative of three independent experiments. *, *p* < 0.05; **, *p* < 0.01.

To determine the physiological relevance of intercellular Nef transfer, we next determined whether intercellular Nef transfer occurred in the context of HIV infection. Jurkat were first infected with HIV-1 NL4-3. NL4-3 ΔNef was included as a control. When the infection efficiency reached about 50%, the cells were stained for HIV-1 Nef and p24 and analyzed for cells that were not infected with HIV but positive for Nef, i.e., p24-Nef+ cells by flow cytometry. Compared to the mock control, there were about 2% p24-Nef+ cells in NL4-3-infected cells and none in NL4-3 ΔNef-infected cells (Fig 2A and 2B, S2A Fig), indicating intercellular Nef transfer in the context of HIV-1 infection. The Nef+p24- cells in the HIV-infected cells could result from intercellular Nef transfer between HIV-infected cells and uninfected cells, or from post-entry Nef expression during early stage of HIV replication. To discern those two possibilities, we took advantage of a GFP-based HIV reporter virus NLGi [53, 57], in which GFP gene is inserted in frame at Nef position whereas Nef is translated under the control of an IRES (S3A Fig). GFP expression from NLGi is an indicator of HIV early gene expression and has been used to identify the HIV-infected cells [53, 57]. Thus, detection of Nef+ cells in the GFP- cells would confirm that intercellular Nef transfer occurred between HIV-infected cells and uninfected cells. MT4 were infected with NLGi. NLGi ΔNef was included as a control. NLGi- or NLGi ΔNef-infected MT4 were used as the donor cells, and co-cultured with NLGi-refractory Jurkat [53, 57] at 1:1 ratio for 48 hr. As expected, about 50% of the co-cultured cells remained uninfected 48 hr post co-culture, determined by GFP expression (S3B Fig). Nef staining showed about 3% Nef+GFP- cells in the NLGi-infected MT4/Jurkat co-culture compared to the mock control and the NLGi ΔNef-infected MT4/Jurkat co-culture (Fig 2C and 2D, S3C Fig), confirming Nef transfer from HIV-infected cells into uninfected cells. There was slightly more Nef transfer detected with NLGi-infected MT4 as the donor cells (Fig 2C and 2D) than with NL4-3-infected MT4 as the donors (Fig 2A and 2B). This was likely due to different levels of Nef expression between the cells infected with those two viruses (S2C Fig).

### Requirement of membrane localization for intercellular Nef transfer

To further determine the specificity of intercellular Nef transfer, we examined the relationship between Nef localization in plasma membrane and its intercellular transfer. Nef is myristoylated on its second amino acid residue glycine, which is important for Nef localization in the plasma membrane and its function [58, 59]. Thus, we performed the intercellular Nef transfer assay using a HIV with the mutation from glycine to alanine (G2A) at the second amino acid residue of Nef. Compared to the wild-type counterpart, G2A mutation led to significant decreases in the percentage of Nef+p24-, namely the efficiency of intercellular Nef transfer (Fig 2E and 2F, S2B Fig). In addition, we also determined the relationship between intercellular Nef transfer and the cell density of the co-culture and the relationship between intercellular Nef transfer and the ratio of the donor cells to target cells in the co-culture. The percentage of the Nef+p24- cells showed gradual increases over the cell density (Fig 2G), or increased ratios of the donor cells to target cells (data not shown). These results demonstrated that Nef membrane localization was important for intercellular Nef transfer and suggest that cell-cell contact is required for this process.

### Extracellular structures associated with intercellular Nef transfer

**Fig 3:**
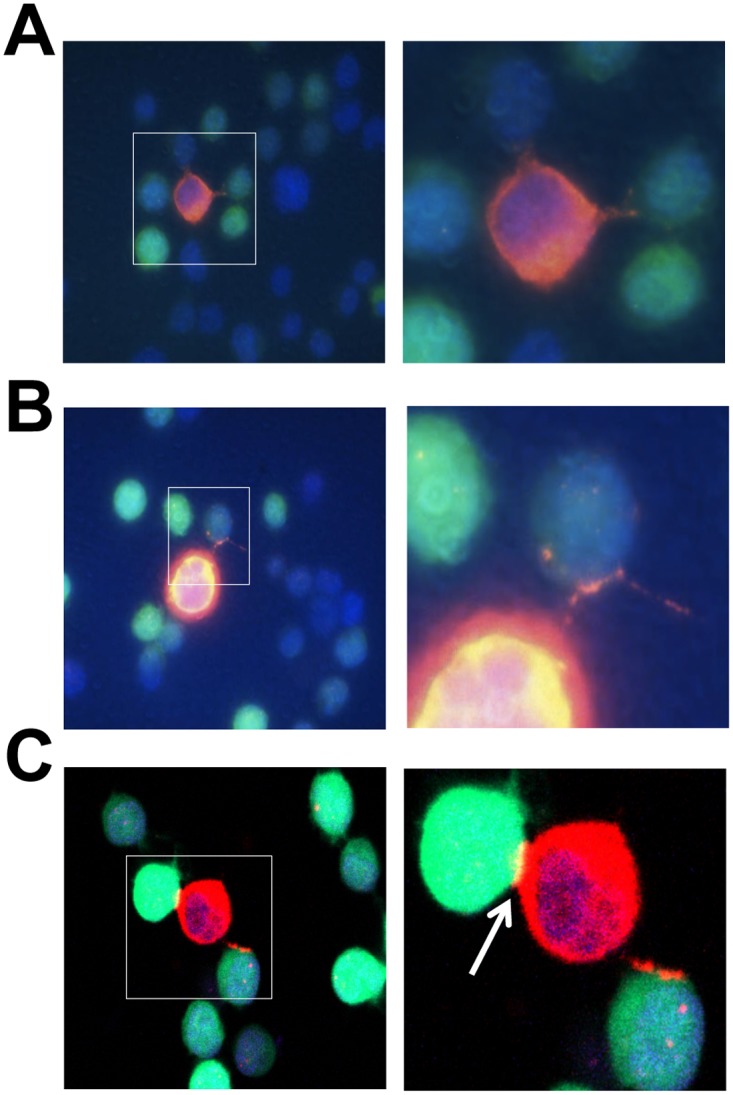
Structures for intercellular Nef transfer. Jurkat were infected with HIV-1 NL4-3 and used to co-culture with GFP-expressing Jurkat (0.5 x 10^6^ each) in a volume of 500 μl medium in a 24-well plate (i.e., at a cell density of 1 x 10^6^/ml) for 16 hr. At the end of the co-culture, the cells were stained using a mouse anti-HIV-1 Nef antibody (sc-65904) and Alexa 555 anti-mouse IgG and analyzed by microscopic imaging. DAPI staining was performed for nuclei. A common type of structures associated with intercellular Nef transfer, i.e., tunneling nanotube formation was noted (**A-C**). Rhodamine channel exposure time of each image was optimized for fine TNT structure, which led to saturated fluorescence signal (p24) in infected cells. Area of interest was marked (left) and its micrograph was taken using a higher mag objective (right). Trogocytosis, also known as virological synapse formed between HIV-infected cells and uninfected cells, was shown by an arrow (**C**).

As discussed above, intercellular protein transfer could be cell-cell contact-dependent such as tunneling nanotubes and trogocytosis or cell-cell contact-independent such as MV or exosomes [3–5]. To discern these two intercellular Nef transfer mechanisms, NL4-3-infected Jurkat and GFP-expressing Jurkat were used in the co-culture assay as the donor and target cells, respectively. Immunostaining of the cells in the co-cultures using an anti-HIV-1 Nef antibody in combination with microscopic imaging confirmed the presence of HIV-1 Nef protein in GFP-expressing Jurkat (Fig 3A–3C). Meanwhile, tunneling nanotubes were noted to form between NL4-3-infected Jurkat and GFP-expressing Jurkat (Fig 3A and 3B). In addition, formation of virological synapse was also noted at the close contact between NL4-3-infectd Jurkat and GFP-expressing Jurkat (Fig 3C, arrow), a typical type of trogocytosis often detected between HIV-infected and uninfected cells [60–62]. These results further indicate that cell-cell contact is the likely mechanism for intercellular Nef transfer.

### Nef was not detected in crude exosomes from Nef-expressing Jurkat

**Fig 4:**
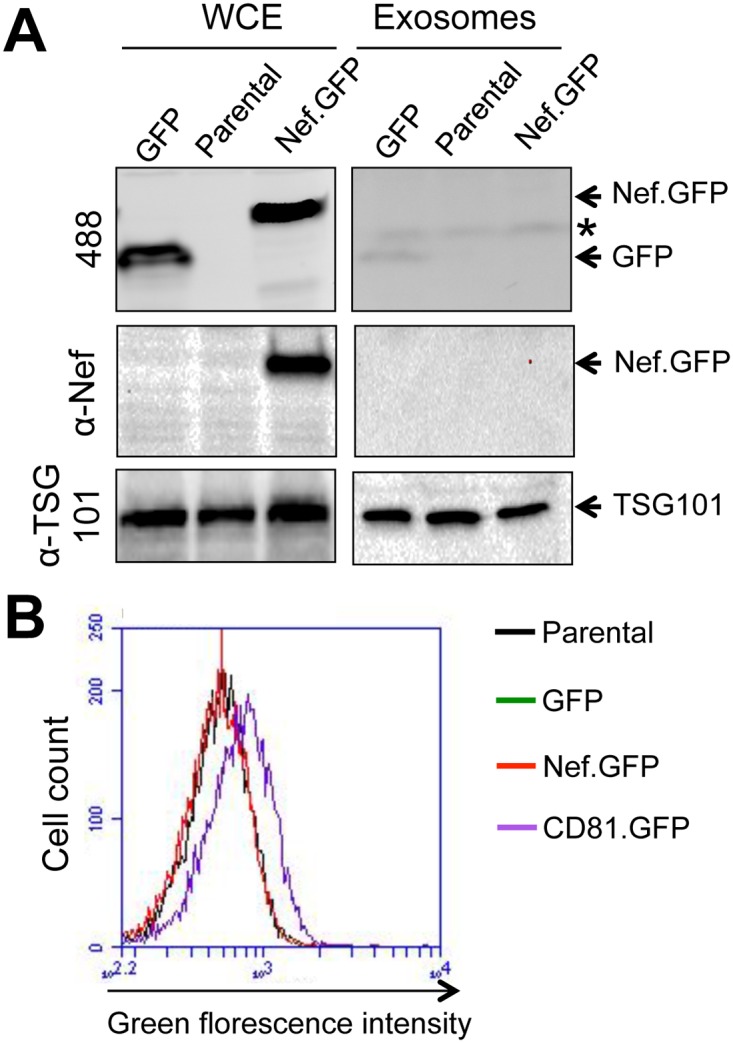
No Nef detection in crude exosomes and no Nef transfer through exosomes from Nef-expressing Jurkat. **A.** Crude exosomes were isolated from GFP- and Nef.GFP-expressing Jurkat, as well as its parental Jurkat by sequential centrifugation. Whole cell lysates (WCE) were also prepared from the cells. Both crude exosomes and WCE were analyzed by Western blotting, followed by direct visualization of the blots at a wavelength of 488 nm or antibody staining and ECL. Samples were subjected to incubation at 37°C for 30 min before SDS-PAGE to facilitate membranous protein detection and GFP visualization. * Non-specific bands. **B.** Fresh Jurkat (1 x 10^5^) were incubated with each of crude exosomes prepared above for 24 hr. The cells were then washed with PBS for multiple times and analyzed for GFP by flow cytometry. Crude exosomes from CD81.GFP-expressing cells were included as a positive control. The data were representative of two independent experiments.

Several studies have reported detection of extracellular Nef *in vitro* [63, 64] and *in vivo* [65]. As discussed in the Introduction, TNT, MV, and exosomes have all been shown to be involved in intercellular Nef transfer [40–46]. Thus, we next examined the roles of cell-cell contact-independent mechanisms such as MV or exosomes in intercellular Nef transfer. To this end, we first tested if Nef would be present in crude exosomes from Nef.GFP-expressing Jurkat. Parental Jurkat and GFP-expressing Jurkat were included as the controls. Crude exosomes were isolated from cell culture supernatants after removal of cells and cell debris and analyzed for Nef presence using Western blotting. GFP and Nef.GFP expression in respective cells was confirmed by direct visualization of GFP (Fig 4A, top panel) or using anti-Nef antibody (Fig 4A, middle panel). However, only GFP but no Nef.GFP was detected in exosomes from those cells. Detection of GFP in the crude exosomes suggests possible contamination of crude exosomes with cell debris or non-specific incorporation of GFP into exosomes. Next, we determined, if any, Nef uptake into Jurkat through exosomes. The crude exosomes prepared from above were added into fresh Jurkat and incubated for 24 hr. Exosomes from CD81.GFP-expressing cells were also used as a positive control, as CD81 is an exosome marker [66–68]. The Jurkat were harvested and analyzed for Nef uptake using flow cytometry. Only incubation with exosomes from CD81.GFP-expressing cells gave rise to GFP detection in Jurkat (Fig 4B). These results together suggest that exosomes may not be significantly involved in intercellular Nef transfer.

### Nef was not detected in the AChE+ fractions of exosomes from HIV-infected Jurkat

**Fig 5:**
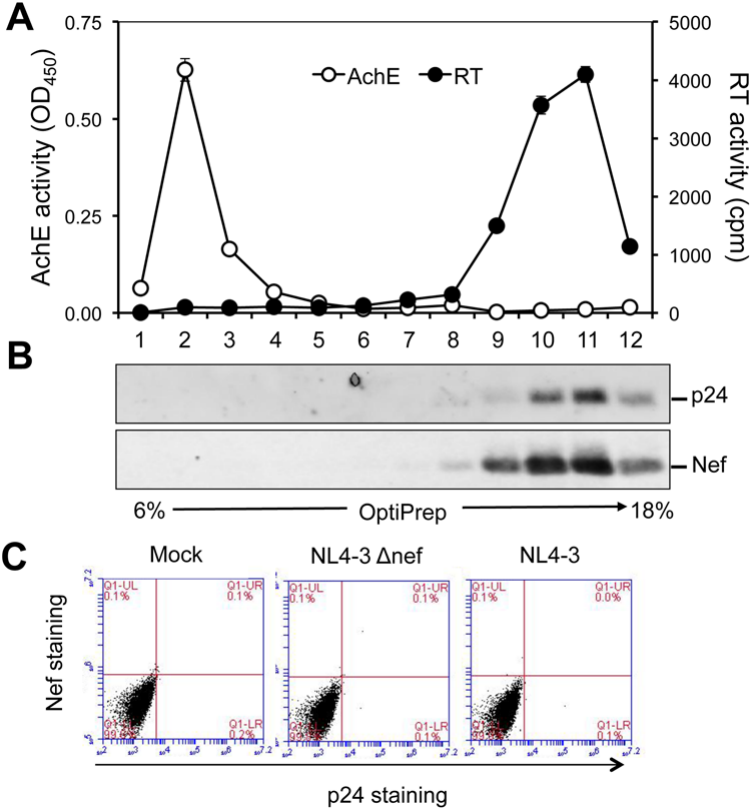
No Nef detection in crude exosomes and no Nef transfer through exosomes from HIV-infected Jurkat. NL4-3-infected Jurkat (10 x 10^6^, about 70% p24+ determined by flow cytometry) were cultured in 20 ml exosome-free medium for 3 days. The culture medium was collected and removed of cell debris by three consecutive steps of centrifugation: 600 *g* for 10 min, 2000 *g*, 10 min, and 10000 *g* for 30 min. The cleared supernatants were then spun at 100,000 *g* for 70 min to obtain exosome pellets. The pellets were suspended in 500 μl PBS and saved as crude exosomes. The crude exosomes were further fractionated in a 5 ml 6–18% OptiPrep gradient at 250000 *g* for 90 min. Following the gradient centrifugation, a total of 12 fractions, 450 μl each from top to bottom was collected. Aliquots of each fraction were used for AChE activity assay (24 μl, open circle, **A**), the reverse transcriptase (RT) activity assay (200 μl, closed circle, **A**), and TCA precipitation and Western blotting using an anti-p24 or anti-Nef antibody (sc-65904) (226 μl, **B**). The AChE and RT activities were mean ± SD of triplicates; the data were representative of three independent experiments. **C.** Culture supernatants were collected from NL4-3-infected, *nef*-deleted NL4-3 (ΔNef)-infected or mock, removed of cells and cell debris, and incubated with fresh Jurkat for 24 hr. At the end of incubation, the cells were double stained using a rabbit anti-HIV-1 Nef antibody, followed by Alexa 488-conjugated anti-rabbit antibody and PE-conjugated mouse anti-p24 antibody. The cells were analyzed for Nef+p24- cells by flow cytometry. The data were representative of triplicate samples and two independent experiments.

HIV-1 Nef has been shown to be selectively packaged into HIV virions [69–71]. Several studies have shown that the multiple rounds of sequential centrifugation-based exosome isolation method as described in the Material and Methods section allows successful separation of exosomes from HIV [42, 43, 72–74]. Thus, to further examine the relationship between HIV-1 Nef and exosomes, we determined whether HIV-1 Nef was present in the exosomes directly derived from HIV-infected CD4 T lymphocytes. Jurkat were infected with HIV-1 NL4-3. The culture supernatants were collected when the infection efficiency reached about 70%, determined by p24 staining and flow cytometry, and little cell death was detected, determined by trypan blue staining (data not shown). The culture supernatants were then subjected to four rounds of sequential centrifugation to ensure complete removal of dead cells and cell debris and then used to obtain the crude exosomes. The crude exosomes were further fractionated in a 6–18% OptiPrep gradient. A total of 12 fractions from the top to the bottom were collected and assayed for the acetylcholinesterase (AChE) activity for exosomes and the RT activity for HIV-1. Consistent with previous findings [42, 72, 74], AChE activity was detected in fraction 1–4, while RT activity was detected in fraction 8–12 (Fig 5A). Meanwhile, the fractions were assayed for HIV-1 p24 and Nef protein by Western blotting. In agreement with the RT activity above, both p24 and Nef were detected in fraction 8–12 (Fig 5B). However, there was little Nef detection in fraction 1–4. Use of crude exosomes derived from 10 times more culture supernatants of HIV-infected Jurkat also did not lead to Nef detection in fractions 1–4 (data not shown). These results indicate that HIV-1 Nef is not present in the AChE+ fractions of exosomes derived from HIV-infected cells. To further determine if exosomes were involved in intercellular Nef transfer in the context of HIV infection, we prepared cell-free culture supernatants from NL4-3-infceted Jurkat and incubated them with fresh Jurkat for 24 hr. Culture supernatants from mock and NL4-3 ΔNef-infected Jurkat were included as the controls. The Jurkat were harvested and stained for Nef and p24. There were no Nef+p24- cells detected in any fresh Jurkat (Fig 5C). These results show that exosomes may not be significantly involved in intercellular Nef transfer in the context of HIV infection.

### Exposure of crude exosomes from Nef-transfected 293T led to little Nef uptake

**Fig 6:**
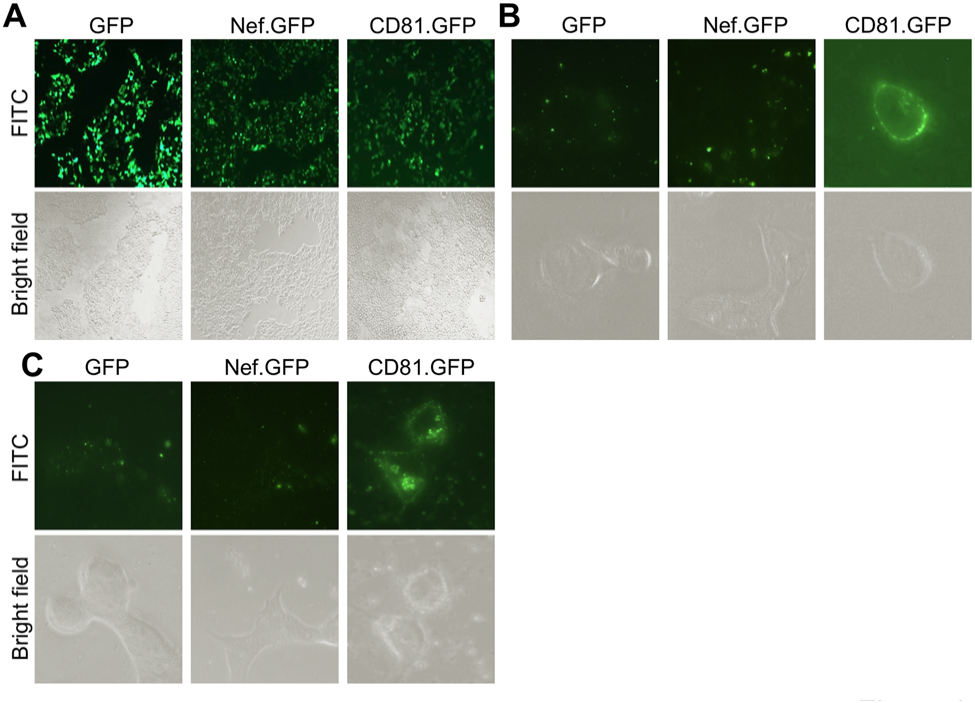
No Nef uptake into 293T by microscopic imaging. 293T (2 x 10^6^) were plated at a 10 cm plate, transfected with GFP, CD81.GFP, or Nef.GFP plasmid, and cultured for 16 hr, followed by direct microscopic imaging with a FITC filter or under the bright field (a 10X objective) (**A**). Transfected cells were then cultured in exosome-free medium for 3 days. Crude exosomes from the culture medium (40 ml) were prepared as described above, suspended in exosome-free medium, added onto fresh 293T in a polylysine-treated glass bottom dish, and incubated for 3 hr (**B**) and 12 hr (**C**). At the end of each incubation, images of the target cells were taken with a FITC filter or under the bright field (a 100X objective). The micrographs were representative of images from multiple fields of two independent experiments.

**Fig 7:**
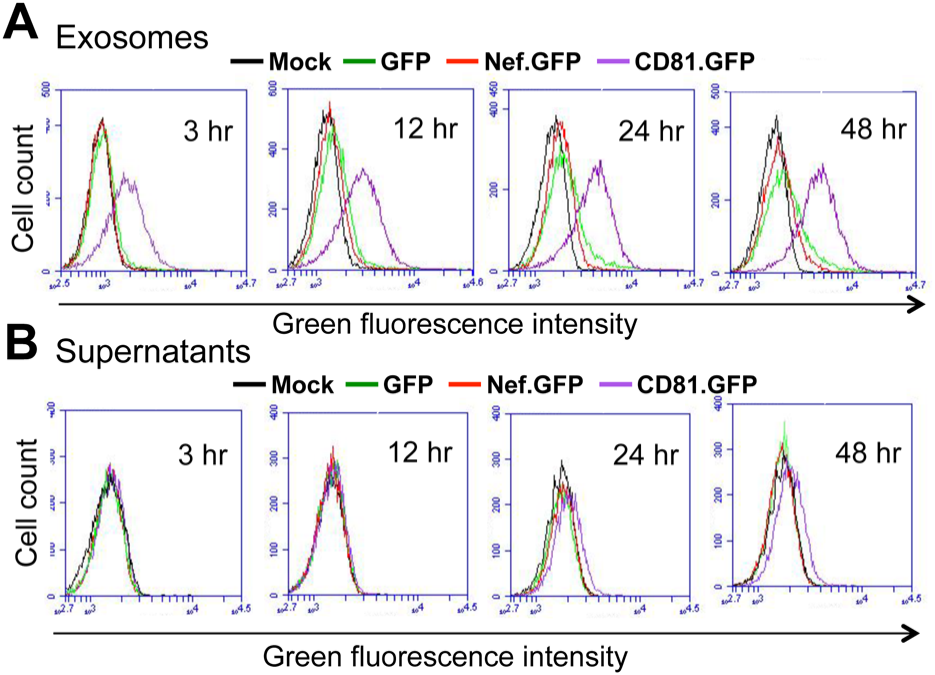
No Nef uptake into Jurkat by flow cytometry. 293T cells were transfected with cDNA3 (Mock), GFP, Nef.GFP, or CD81-GFP plasmid and cultured in exosome-free medium for 3 days. Cell culture media were collected and removed of cell debris (Supernatants), or used to prepare crude exosomes (Exosomes) as described above. Jurkat (1 x 10^5^) were incubated with 100 μl crude exosomes (**A**) or 100 μl supernatants (**B**) for 3, 12, 24, or 48 hr. The cells were then washed with PBS multiple times and analyzed by flow cytometry. The data were representative of four independent experiments.

Earlier studies have detected Nef in exosomes from Nef-transfected cells [42, 44]. To determine if Nef could be taken up through crude exosomes from Nef-transfected cells, we transfected 293T with Nef.GFP-expressing plasmid. GFP and CD81.GFP were included as a negative and positive control, respectively. The transfection efficiency was determined to be comparable and over 90% among these three transfections (Fig 6A). The cell culture media were collected and removed of dead cells and cell debris. The supernatants were used to isolate crude exosomes. Fresh 293T were incubated with the crude exosomes and monitored for cellular uptake of GFP, Nef.GFP, and CD81.GFP by microscopic imaging. As expected, treatment of fresh 293T with crude exosomes isolated from CD81.GFP-transfected cells showed uptake and membrane localization of CD81.GFP (Fig 6B) and increased accumulation of CD81.GFP on the plasma membrane and cytoplasm over time (Fig 6C). However, treatment of fresh 293T with the crude exosomes from Nef.GFP transfected cells showed a nonspecific and background GFP pattern similarly to those treated with the crude exosomes from GFP-transfected cells at both indicated time points. To accurately and quantitatively determine possible Nef uptake, fresh Jurkat were incubated with the same crude exosomes and respective supernatants for up to 48 hr, the cellular uptake of GFP, Nef.GFP and CD81.GFP was determined by flow cytometry. Similarly, CD81.GFP showed uptake beginning at 3 hr and gradual increases up to 48 hr in cells treated with the crude exosomes from CD81.GFP-transfected cells (Fig 7A). Compared to the crude exosomes from mock transfection, there appeared slight but similar uptake of GFP and Nef.GFP by Jurkat from respective crude exosomes. In addition, treatment of fresh Jurkat with the supernatant from CD81.GFP-transfected cells only showed slight CD81.GFP uptake at 24 hr and more CD81.GFP uptake at 48 hr (Fig 7B). Compared to the mock control, little GFP or Nef.GFP uptake was detected in Jurkat treated with the supernatants from GFP- or Nef.GFP-transfected cells. Taken together, these results further indicate that MV and exosomes are unlikely involved in intercellular Nef transfer.

### Nef was detected in the AChE+ fractions from Nef.GFP-transfected 293T

**Fig 8:**
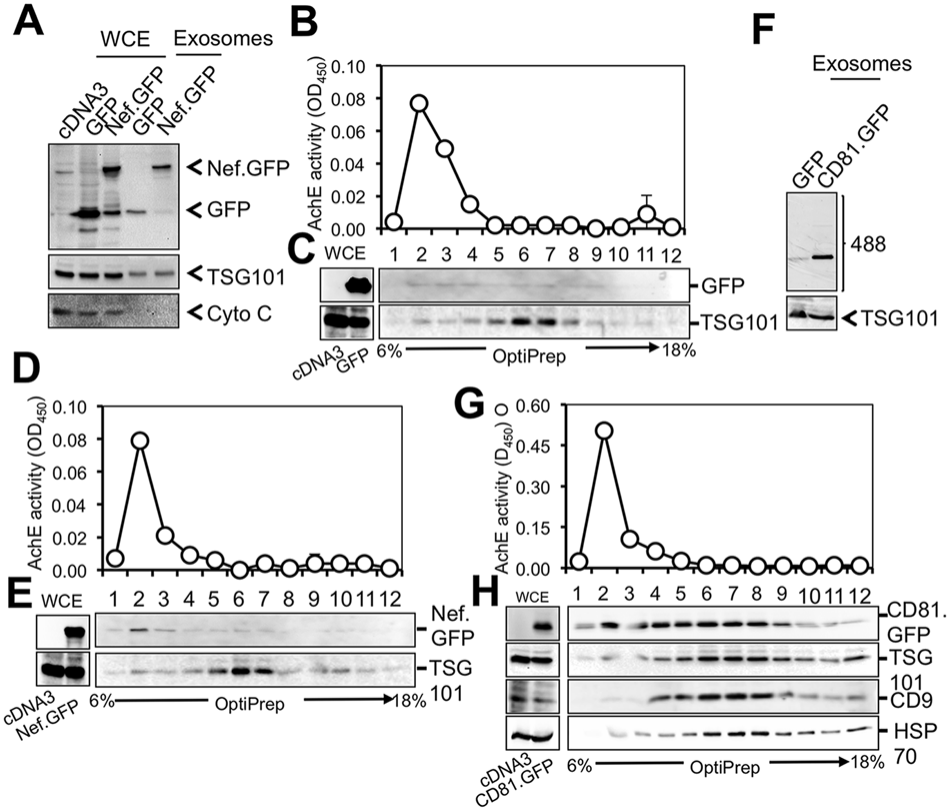
Nef detection in the AChE+ fractions from Nef-transfected 293T. 293T (2 x 10^6^) were plated in a 10 cm plate and transfected with GFP (**A-C**), Nef.GFP (**A, D & E**), or CD81.GFP (**F-H**). Transfected cells were then cultured in exosome-free medium for 3 days. Culture medium was collected and pooled (about 70 ml total) for crude exosomes (500 μl) as described above, while cells were harvested for cell lysates for whole cell extracts (WCE). WCE and crude exosomes were analyzed by Western blotting using TSG101 and cytochrome C (cyto C) antibody (**A**). GFP and Nef.GFP and CD81.GFP were visualized at a wavelength of 488 nm (**A & F**). The data were representative of three independent experiments. Then the crude exosomes were subjected to the OptiPrep gradient centrifugation and fractionated. Aliquots of each fraction were used for AChE activity assay (24 μl, **B, D & G**). The remaining fractions were diluted in 4 ml PBS and spun at 100,000 *g*, 70 min. The pellets were lysed in the RIPA buffer followed by Western blotting using indicated antibodies (**C, E & H**). WCE (100 μg) were included as controls (**C, E & H**). The AChE activities were mean ± SD of triplicates; the data were representative of three independent experiments.

To further address the possibility of Nef incorporation into exosomes, we next determined whether Nef was present in crude exosomes from Nef-transfected cells. We transfected 293T with Nef.GFP-expressing plasmid, as well as GFP and CD81.GFP plasmids. Crude exosomes were prepared and analyzed by Western blotting. Cells were harvested and also analyzed by Western blotting. GFP and Nef.GFP were detected in whole cell lysates as well as in crude exosomes (Fig 8A). Absence of cytochrome C detection in the exosomes ruled out the possibility of cells and cell debris in the exosomes. Thus, detection of GFP and Nef.GFP in exosomes is likely an artifact of a high transfection efficiency and over-expression of both proteins in 293T. To further determine Nef association with exosomes, crude exosomes from above were fractionated through the 6–18% OptiPrep gradient. A total of 12 fractions from top to the bottom were collected for the AChE activity assay and Western blotting. TSG101 was used as an exosome marker [75] for Western blotting. For GFP crude exosomes, the AChE activity was detected in fractions 1–4 (Fig 8B). A trace amount of GFP was detected in most fractions. Interestingly, TSG101 was detected slightly higher in fraction 2 and much higher in fractions 5–8 (Fig 8C). For Nef.GFP exosomes, the AChE activity was detected in fractions 1–4 (Fig 8D) and TSG101 was detected slightly higher in fraction 2 and much higher in fractions 5–8 (Fig 8E). In addition, Nef.GFP was clearly detected in fraction 2 and 3, seemingly only co-existent with AChE+ fractions. For CD81.GFP crude exosomes, CD81.GFP and GFP were detected in crude exosomes (Fig 8F). The AChE activity was similarly detected in fractions 1–4 (Fig 8G). Unlike Nef.GFP (Fig 8C), CD81.GFP was detected not only in fraction 2 and co-existent with AChE+ fractions, but also in fractions 4–9 and co-existent with TSG101+ fractions (Fig 8H). Besides CD81 and TSG101, a trace amount of two additional exosome markers CD9 and HSP70 [75] were detected in fractions 2 and much more in fraction 4–9. Taken together, these results show that this exosome isolation protocol successfully separated crude exosomes into two groups: AChE+CD81^low^/TSG101^low^ exosomes and AChE- CD81^high^/TSG101^high^ exosomes and that Nef was detected only in AChE+CD81^low^/TSG101^low^ exosomes from Nef-GFP-transfected cells.

### Sensitivity of AChE-/CD81^high^/TSG101^high^ exosomes to detergent treatment

**Fig 9:**
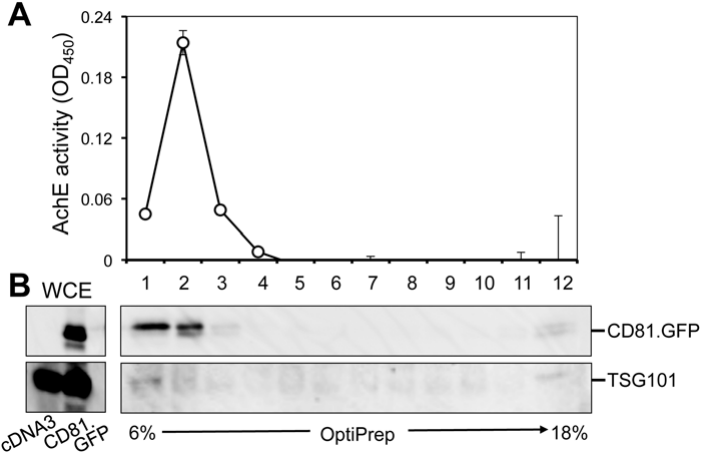
Detection of CD81.GFP in the AChE+ fractions derived the crude exosomes treated with RIPA buffer. 293T (2 x 10^6^) were plated in a 10 cm plate and transfected with CD81.GFP. Transfected cells were cultured in exosome-free medium for 3 days. Culture medium was collected, pooled (about 30 ml) and used to isolate crude exosomes as described above. The crude exosome pellet was lysed in 100 μl RIPA buffer, subjected to 3 rounds of freezing and thawing on dry ice, and diluted in 400 μl PBS, followed by the same 6%-18% OptiPrep gradient centrifugation. Aliquots of each fraction were used for AChE activity assay (24 μl, **A**). The remaining fractions (426 μl) were diluted in 4 ml PBS and spun by 100,000 *g*, 70 min. The pellets were lysed in the RIPA buffer, followed by Western blotting using an anti-TSG101, or CD81.GFP antibody. The AChE activities were mean ± SD of triplicates; the data were representative of three independent experiments.

Distinct levels of exosomal markers AChE, CD81, TSG101, CD9, and HSP70 between AChE+/CD81^low^/TSG101^low^ exosomes and AChE-/CD81^high^/TSG101^high^ exosomes suggest the existence of two types of exosomes. To confirm the membrane vesicular structure of these two exosome subpopulations, we next analyzed the sensitivity of these two types of exosomes to detergent treatment. To this end, we suspended the crude exosomes from CD81.GFP-transfected cells with the RIPA buffer containing both nonionic and ionic detergents Triton X-100, SDS, and DOS, followed by three rounds of freezing and thawing. The RIPA-treated crude exosomes were then subjected to the 6–18% OptiPrep gradient centrifugation. Compared to untreated crude exosomes (Fig 8G and 8H), the AChE activity was still detected in fractions 1–4 but at a much decreased level (Fig 9A); and CD81.GFP and TSG101 were completely absent from fraction 4–8 and were detected in fraction 1 but at a much decreased level (Fig 9B). These results indicate that AChE+/CD81^low^/TSG101^low^ exosomes and AChE-/CD81^high^/TSG101^high^ exosomes are both membrane-bounded vesicles sensitive to detergent treatment. Interestingly, AChE+/CD81^low^/TSG101^low^ showed some resistance to RIPA buffer treatment as they were still detected at the original fractions while the AChE activity showed a reduction by about 50% compared to untreated control (data not shown). This resistance is possibly due to the difference in their lipid contents in their outer membranes such as lipid raft enrichment that could be resistant to the nonionic detergent such as Triton X-100, the major component in RIPA buffer. These results may suggest that these two populations of exosomes differ in their biogenesis pathways.

### Intracellular and extracellular Nef localization

**Fig 10:**
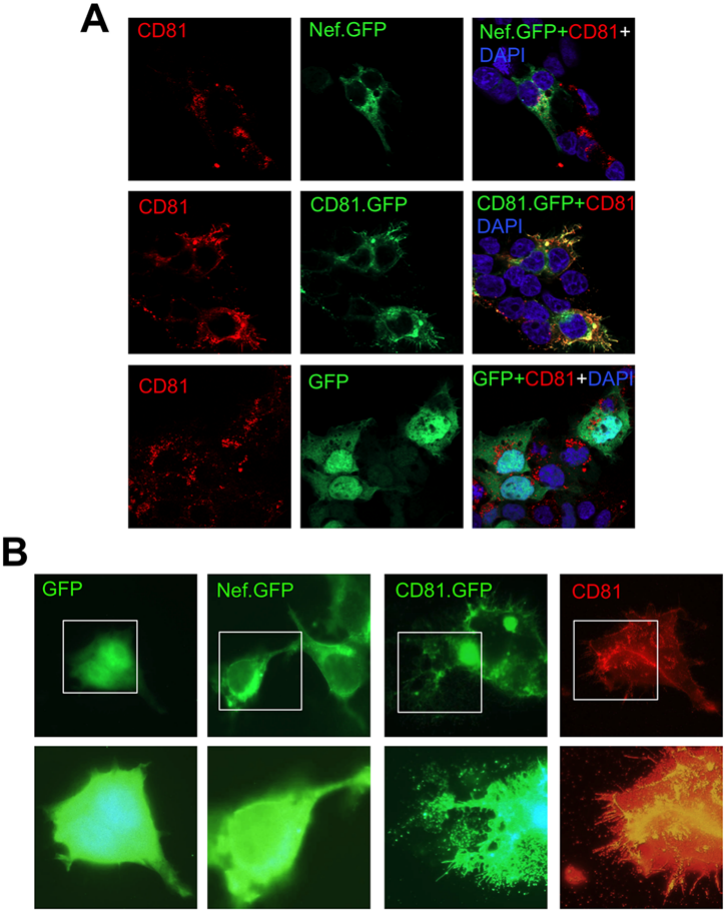
Nef and CD81 localization in intracellular and extracellular vesicles. 293T (5 X 10^4^) were plated in a 24-well plate and transfected with GFP, Nef.GFP, or CD81.GFP plasmid. Twenty-four hours post transfection, the cells were re-plated on top of polylysine-treated coverslip in a 24-well plate. Cells were fixed after 24 hr and processed for immunostaining using an anti-CD81 antibody, followed by Alexa Fluor 555-conjugated goat anti-mouse secondary antibody, which allows detection of both endogenous and exogenous CD81 expression and localization using a rhodamine filter under confocal microscope (60X objective). GFP tagged protein expression and localization were detected using a FITC filter. **A.** CD81 Nef co-localization; **B.** Extracellular vesicle-like structures (with extended exposure time). The micrographs were representative of images from multiple fields of three independent experiments.

HIV-1 Nef is myristoylated at its second amino acid glycine and as a result, is targeted to the plasma membrane; but it is also detected in cytosol around the perinuclear region [30, 58, 76, 77]. Exosomal biogenesis involves formation of internal vesicles in the endosomes and release of internal vesicles from multivesicular bodies to the outside of the cell [75, 78]. Thus, to ascertain the relationship between HIV-1 Nef and exosomes, we determined intracellular and extracellular Nef localization in comparison to CD81, the widely used exosomal marker [66–68]. 293T were transfected with GFP, Nef.GFP, or CD81.GFP, followed by immunostaining of endogenous CD81. Intracellular and extracellular localization of endogenous CD81 and Nef.GFP/CD81.GFP proteins were analyzed by confocal microscopic imaging. As expected, GFP was expressed throughout the cell; CD81.GFP was detected in the plasma membrane as well as in exosomal vesicle-like structures in the cell (Fig 10A, S4 Fig and S1 Movie and S2 Movie) and out the cell (Fig 10B). In contrast, Nef was detected on the plasma membrane and cytosol, but not evidently in co-localized with CD81 both in and out the cells. Staining of endogenous CD81 showed similar CD81 localization as CD81.GFP and staining of exogenous CD81.GFP using CD81 antibody further confirmed the specificity of CD81 localization. These results provide additional evidence to suggest that HIV-1 Nef is not likely associated with exosomes.

## Discussion

TNT and MV are two main structures for intercellular protein transfer [2]. TNT are 50–200 nm in diameter and a length of up to several cells and can be formed among a variety of cells [3, 6]. MV can be either shedding vesicles, which are outward blebbing of small vesicles of 100–1000 nm in diameter directly from cellular plasma membrane [79, 80], or exosomes, which are exocytosis of internal luminal vesicles of 30–100 nm in diameter formed in the multivesicular bodies and released from the cells as a result of fusion of multivesicular bodies with plasma membrane [68, 81]. Unlike TNT, protein transfer through exosomes also involves uptake of exosomes by the target cells [81, 82]. Thus, these two intercellular protein transfer mechanisms could be discerned by the presence of TNT as well as by the extent to which exosomes is involved. In this study, we show that Nef is transferred to additional cell types including astrocytes and that the transfer involves TNT formation (Figs 1–3). Given that HIV can be transferred from cell to cell via virological synapse and that Nef is incorporated into the virions, trogocytosis also likely leads to intercellular Nef transfer. Several approaches had been attempted to devise a functional read-out for intercellular Nef transfer, Nef-induced CD4 down-regulation, Nef-induced MHC I down-regulation, Nef effects on HIV-LTR-driven reporter, and Nef-induced cytokine production. However, we have so far been unsuccessful due to the technical difficulties. Moreover, we show that Nef transfer is likely independent of exosomes: (1) there was little Nef uptake in the form of free protein or exosomes in the culture supernatants (Figs 4B, 5C, 6 and 7); (2) Nef was not detected in the exosomes obtained from HIV-infected Jurkat and Nef.GFP expressing Jurkat (Figs 4A and 5); (3) Nef was detected only in a subpopulation of exosomes (see more discussion below) when Nef was ectopically expressed in 293T (Fig 8A–8E); and (4) Nef was not localized in exosomal vesicle-like structures in and out the cell (Fig 10). Thus, we conclude that intercellular Nef transfer is cell-cell contact dependent and may not likely involve exosomes.

There are several lines of evidence to support that Nef is directly involved in TNT formation. TNT formation was first described in 2004 [83]. Besides the morphology, little is known about how TNT formation is regulated. Until recently, M-Sec has been shown to be important for TNT formation through actin cytoskeleton remodeling [84, 85]. Interestingly, HIV-1 Nef has long been linked to actin cytoskeleton remodeling. HIV-1 Nef induces the rearrangement of actin microfilaments, leading to uropod and ruffle formation in dendritic cells [86]. In addition, we have shown that Nef expression is associated with T cell polarization and filopodia formation in T cells [43, 56, 87]). Furthermore, we have recently demonstrated that Nef plays important roles in virological synapse-mediated HIV transfer through regulation of F-actin polymerization and filopodia formation (Green and He, manuscript in revision 2014). TNT formation has been detected to transfer HIV and Nef protein between macrophages and B cells [40]. These findings together support the notion that Nef itself promotes TNT formation to facilitate its own intercellular transfer and may also explain the less target cell type-dependent nature of Nef targeting. Lastly, M-Sec is mainly expressed in cells such as dendritic cells, macrophages and specialized enterocytes M cells, and in lymphoid tissues such as fetal liver and spleen [84]. It would be very interesting to determine whether M-Sec is involved in Nef-induced TNT formation and whether M-Sec plays any roles in intercellular Nef transfer from HIV-infected cells to bystander cells.

Using three independent approaches, we were not able to detect Nef association with exosomes. Those approaches are exosome uptake (Figs 4B, 5C, 6 and 7); purification of exosomes from HIV-infected and Nef.GFP-expressing Jurkat (Figs 4A and 5); and microscopic imaging (Fig 10). Nef has been detected in the crude vesicle preparations and exosomes [41, 42, 44–46, 88]. Consistent with those findings, we have shown Nef detection in crude exosomes and AChE+ exosomes from Nef-transfected 293T (Fig 8A–8E) or HIV-transfected 293T (S5 Fig). In one of the early studies, Nef has also been detected in AChE+ exosomes [42]. Also, it is important to note that Nef has been shown to promote massive MV shedding from the plasma membrane of Nef-producing cells [41]. Thus, it is possible that Nef detection in the exosomes is a combined result of Nef over-expression, Nef-increased MV shedding from plasma membrane, exosomes of less purity, and the inability of the size-based exosome purification protocol to separate plasma membrane-derived shedding vesicles from MVB-derived exosomes. In addition, detection of a low level of GFP in the exosome uptake assay (Figs 6 and 7) and in the purified exosomes (Figs 4A, 8A and 8F) suggests that there is some non-specific artifact when the ectopic protein expression strategy is exploited. Moreover, in agreement with an early study demonstrating that purified exosomes from HIV-infected cells contains little Nef by proteomic analysis [89], we have not been able to detect Nef in exosomes from HIV-infected Jurkat (Fig 5). Taken together, Nef detection in the exosomes from Nef-transfected cells should be dealt with caution.

The exosome purification protocol has led to successful separation of exosomes into two populations (Fig 8): the first one is AChE+/CD81^low^/TSG101^low^ exosomes, and the second one is AChE-/CD81^high^/TSG101^high^ exosomes. The first group has the calculated OptiPrep density of 6.0–9.3%, which has been confirmed to be exosomes by electron transmission microscopic imaging [90]; the second group has the OptiPrep density of 9.3%- 14.7%, which corresponds to the exosome fractions reported by many other groups, e.g., [45, 72]. In addition, we showed that the both population was sensitive to RIPA treatment (Fig 9). Interestingly, we noticed that the AChE+/CD81^low^/TSG101^low^ exosomes showed some resistance to the detergent treatment while the AChE-/CD81^high^/TSG101^high^ exosomes were completely removed from its original fractions. It is known that GPI anchored proteins such as AChE are concentrated in lipid raft domains of plasma membrane and exosomes [91, 92]. On the other hand, lipid rafts are insoluble in nonionic detergent Triton-X100 and often detected in low buoyant density on sucrose density gradients [93]. Since our protocol ensures the removal of large plasma membrane shedding debris, we believe that the unexpected detergent resistance of AChE+/CD81^low^/TSG101^low^ exosomes is due to enrichment of lipid raft on its surface and that Nef is associated with exosomal lipid rafts. However, other possibilities such as presence of non-exosomal membrane structures cannot be excluded. Taken together, these results suggest the likely existence of two types of exosomes.

## Supporting Information

S1 Fig Intercellular HIV-1 Nef transfer from Nef expressing Jurkat to HPA. Nef.GFP-expressing Jurkat (0.5 x 10^6^) were co-cultured with 0.5 x 10^6^ of HPA in a volume of 200 μl medium in a 24-well plate (i.e., at a cell density of 0.5 x 10^6^/ml) for 16 hr (top panels). GFP-expressing Jurkat were included as a control (bottom panels). HPA were identified by GFAP staining, and DAPI staining was also performed to discern HPA from Nef.GFP-expressing Jurkat by the size of the nuclei. Nef transfer from Nef.GFP-expressing Jurkat to HPA was shown by arrows.
(TIF)

S2 Fig Dot plots of HIV-1 Nef transfer from HIV-infected cells to bystander cells. Dot plots for Fig 2. **A.** HIV-1 NL4-3 (Wt) and *nef*-deleted NL4-3 (ΔNef) **B.** HIV-1 HXB2 (Wt) and *nef*-myristoylation mutant HXB2 (A2G). **C.** Western blotting for Nef, gag and β-actin.
(TIF)

S3 Fig Intercellular Nef transfer from NLGi infected MT4 to uninfected Jurkat. HIV (NLGi/NLGi ΔNef) infected MT4 were co-cultured with Jurkat for 48 hr (1:1 ratio at total cell density 1 million/ml). Cells are processed to immunostaining for Nef (APC) before analyzed by FACS. **A.** Schematic of NLGi HIV. GFP-IRES-Nef cassette is inserted in frame in replace of the first 34 amino acid HIV Nef gene. It expresses GFP as an indicator of the early gene expression as well as Nef itself. *NLGI ΔNef were obtained by Xhol digestion and filling in the gap using T4 DNA polymerase. **B.** Dot plots of GFP (FL-1) detection of 48 hr co-cultured samples without Nef staining. **C.** Dot plots of Nef staining, GFP detection of 48 hr co-cultured samples.
(TIF)

S4 Fig Intracellular Nef and CD81 localization Z stack and ortho analysis. 293T (5 X 10^4^) were plated in a 24-well plate and transfected with GFP, Nef.GFP, or CD81.GFP plasmid. Twenty-four hours post transfection, the cells were re-plated on top of polylysine treated coverslip in a 24-well plate. Cells were fixed after 24 hr and processed for immunostaining using an anti-CD81 antibody, followed by Alexa Fluor 555-conjugated goat anti-mouse secondary antibody, which allows detection of both endogenous and exogenous CD81 using a rhodamine filter under confocal microscope (60X objective). GFP tagged protein expression and localization were detected using a FITC filter. **A.** Single channel pictures of CD81 staining and GFP expression. **B.** Ortho analysis of the same field. **Attached movies.** Z stack.
(TIF)

S5 Fig Nef detection in AChE+ exosomes and virus fractions from gagi-transfected 293T. 293T (2 x 10^6^) were plated in a 10 cm plate and transfected with gagi, GFP or cDNA3. Transfected cells were then cultured in exosome-free medium for 3 days. Culture medium was collected and pooled (about 70 ml total) for crude exosomes (500 μl) as described above, while cells were harvested for cell lysates. **A.** Crude exosomes (40 μl) were analyzed by Western blotting using anti-TSG101 antibody. GFP and CD81.GFP were visualized at a wavelength of 488 nm. **B & C.** The remaining 460 μl crude exosomes from gagi-transfected cells were loaded on top of 6%-18% OptiPrep gradient centrifugation followed by fractionation as described above. Aliquot of each fraction was used for AChE activity assay (24 μl) (**B**). The remaining sample of each fraction was diluted in 4 ml PBS and spun at 100,000 g, 70 min. The pellets were lysed in the RIPA buffer followed by Western blotting using indicated antibodies (**C**). p24.GFP were visualized by 488 nm detection (**A & C**). The AChE activity was mean ± SD of duplicate samples. The data were representative of three independent experiments.
(TIF)

S1 Movie Z Stack of CD81.GFP imaging. (MOV)

S2 Movie Z Stack of Nef.GFP imaging. (MOV)
