# Supplementary figures and images for: Exosomes Are Unlikely Involved in Intercellular Nef Transfer
Source: PLoS One. 2015 Apr 28;10(4):e0124436. doi: 10.1371/journal.pone.0124436 (PMC4412529; doi:10.1371/journal.pone.0124436)

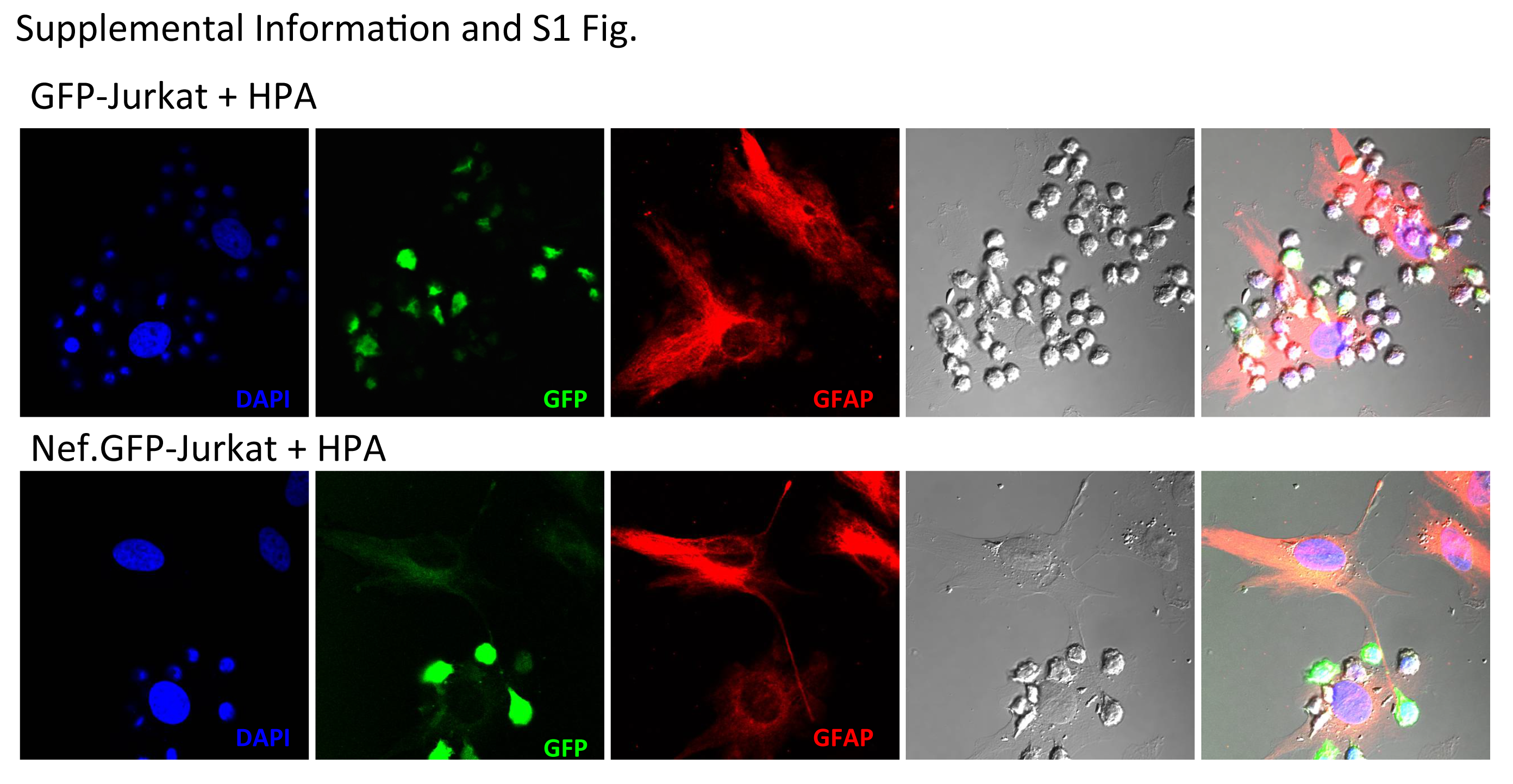

Supplement: S1 Fig — Nef.GFP-expressing Jurkat (0.5 x 10^6^) were co-cultured with 0.5 x 10^6^ of HPA in a volume of 200 μl medium in a 24-well plate (i.e., at a cell density of 0.5 x 10^6^/ml) for 16 hr (top panels). GFP-expressing Jurkat were included as a control (bottom panels). HPA were identified by GFAP staining, and DAPI staining was also performed to discern HPA from Nef.GFP-expressing Jurkat by the size of the nuclei. Nef transfer from Nef.GFP-expressing Jurkat to HPA was shown by arrows. (TIF) [file pone.0124436.s001.tif]

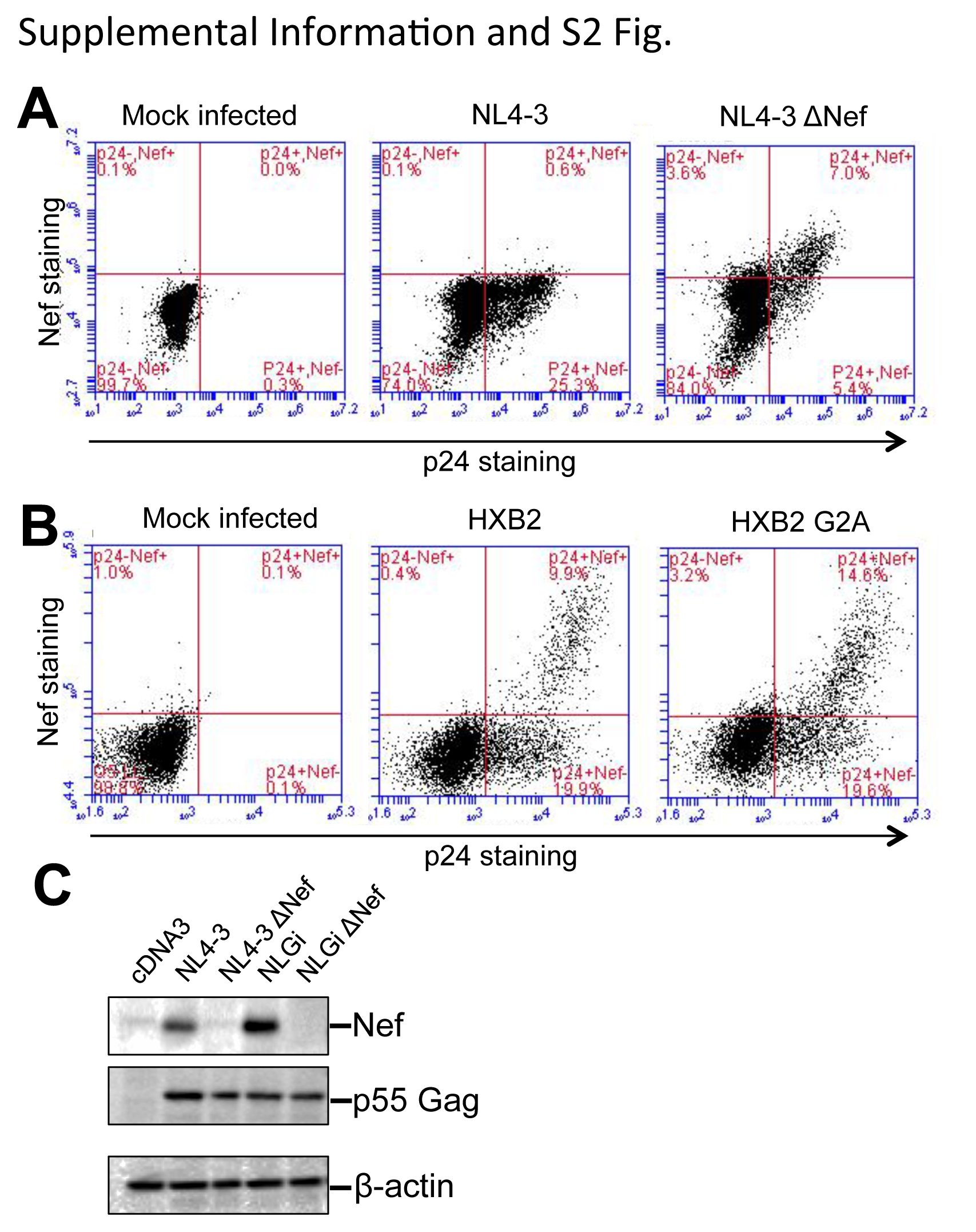

Supplement: S2 Fig — Dot plots for Fig 2. A. HIV-1 NL4-3 (Wt) and nef-deleted NL4-3 (ΔNef) B. HIV-1 HXB2 (Wt) and nef-myristoylation mutant HXB2 (A2G). C. Western blotting for Nef, gag and β-actin. (TIF) [file pone.0124436.s002.tif]

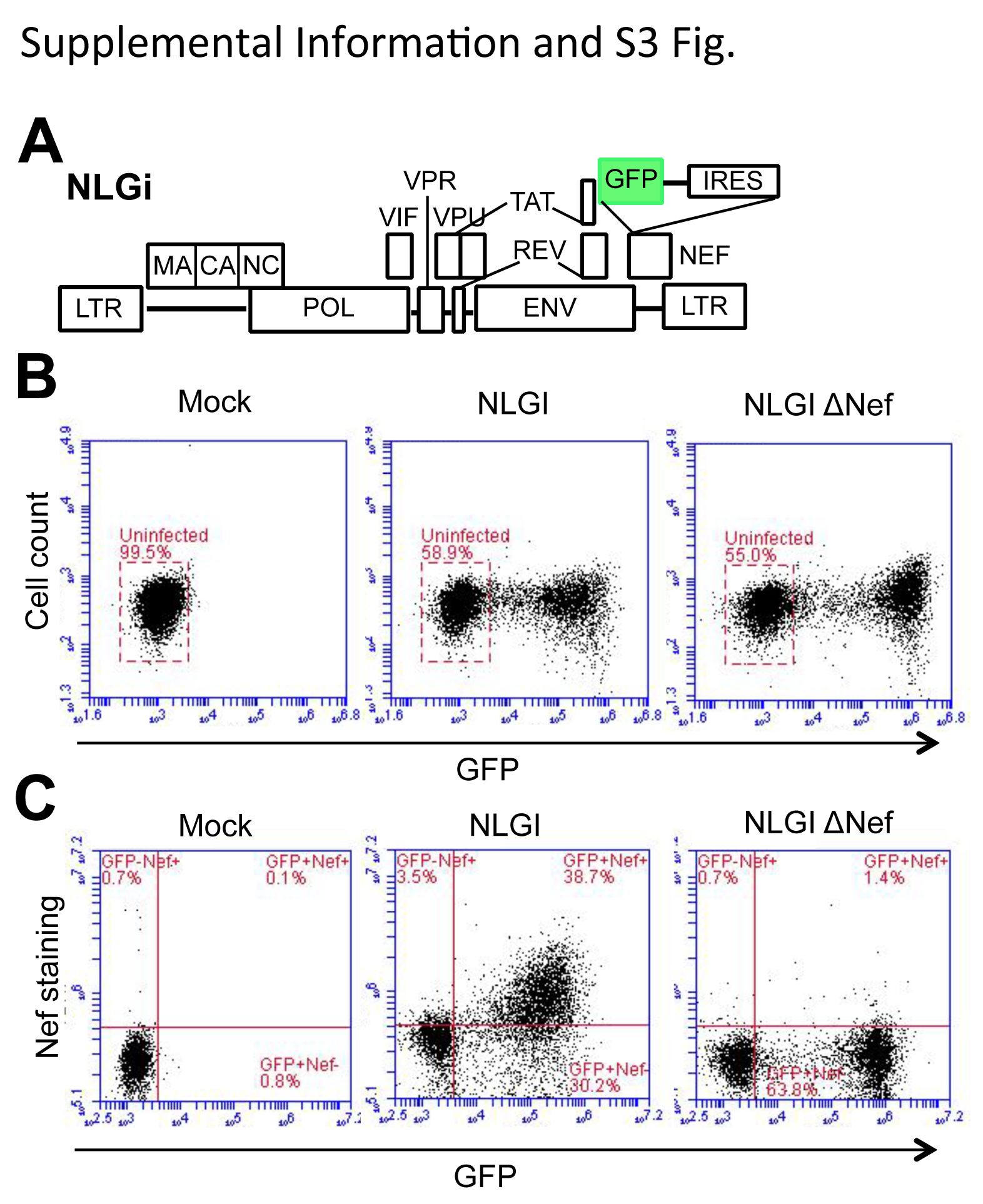

Supplement: S3 Fig — HIV (NLGi/NLGi ΔNef) infected MT4 were co-cultured with Jurkat for 48 hr (1:1 ratio at total cell density 1 million/ml). Cells are processed to immunostaining for Nef (APC) before analyzed by FACS. A. Schematic of NLGi HIV. GFP-IRES-Nef cassette is inserted in frame in replace of the first 34 amino acid HIV Nef gene. It expresses GFP as an indicator of the early gene expression as well as Nef itself. *NLGI ΔNef were obtained by Xhol digestion and filling in the gap using T4 DNA polymerase. B. Dot plots of GFP (FL-1) detection of 48 hr co-cultured samples without Nef staining. C. Dot plots of Nef staining, GFP detection of 48 hr co-cultured samples. (TIF) [file pone.0124436.s003.tif]

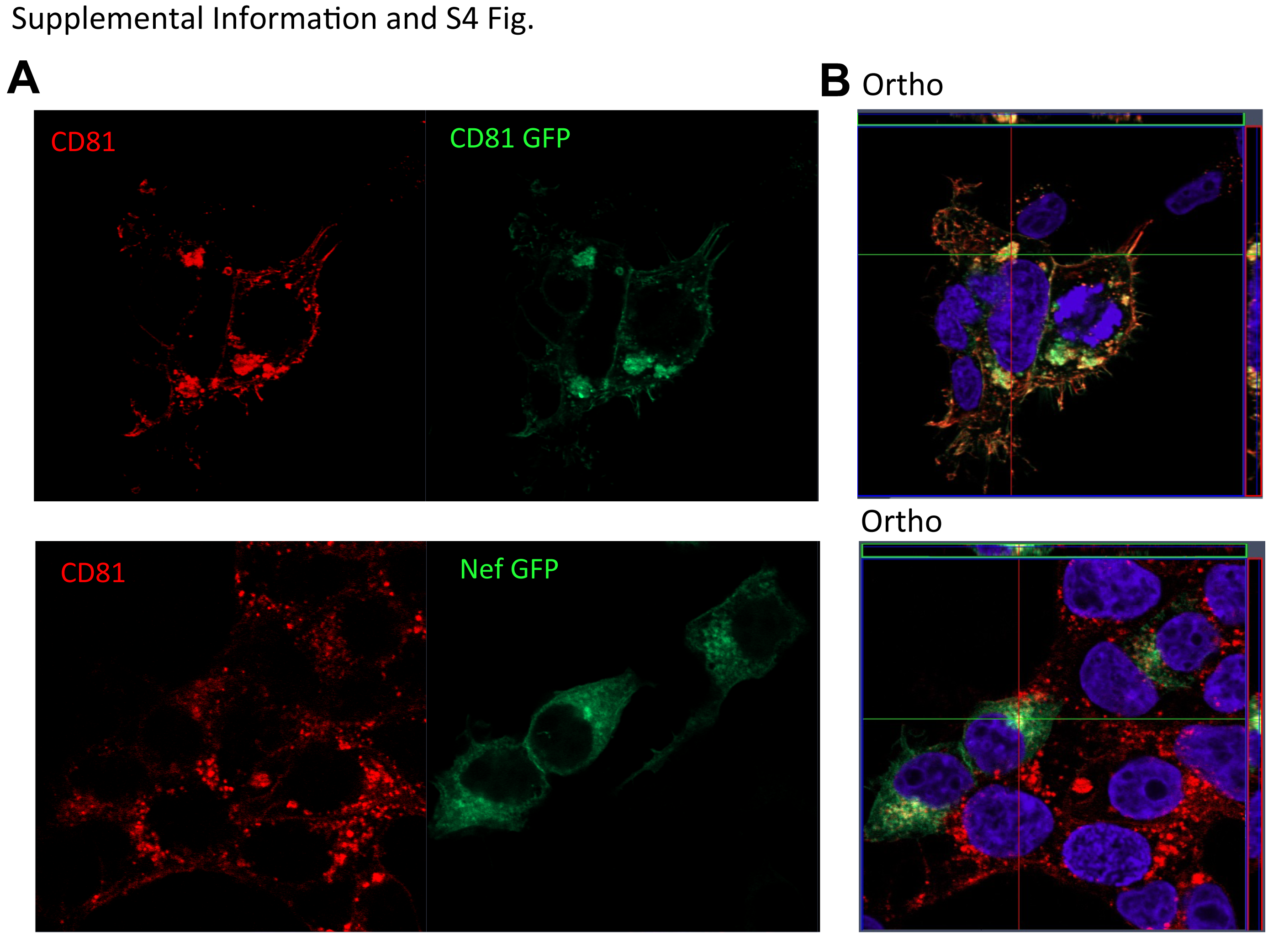

Supplement: S4 Fig — 293T (5 X 104) were plated in a 24-well plate and transfected with GFP, Nef.GFP, or CD81.GFP plasmid. Twenty-four hours post transfection, the cells were re-plated on top of polylysine treated coverslip in a 24-well plate. Cells were fixed after 24 hr and processed for immunostaining using an anti-CD81 antibody, followed by Alexa Fluor 555-conjugated goat anti-mouse secondary antibody, which allows detection of both endogenous and exogenous CD81 using a rhodamine filter under confocal microscope (60X objective). GFP tagged protein expression and localization were detected using a FITC filter. A. Single channel pictures of CD81 staining and GFP expression. B. Ortho analysis of the same field. Attached movies. Z stack. (TIF) [file pone.0124436.s004.tif]

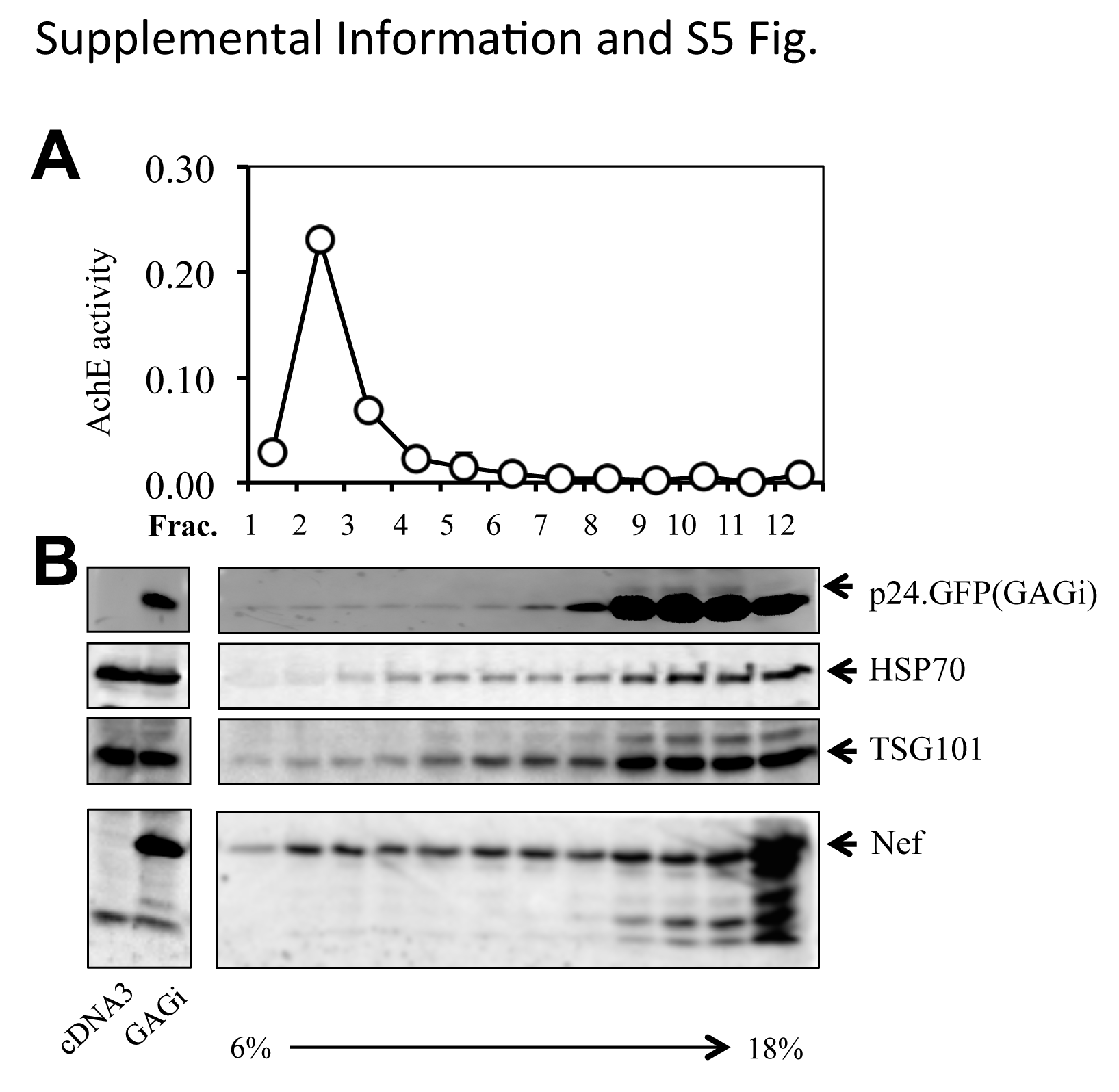

Supplement: S5 Fig — 293T (2 x 10^6^) were plated in a 10 cm plate and transfected with gagi, GFP or cDNA3. Transfected cells were then cultured in exosome-free medium for 3 days. Culture medium was collected and pooled (about 70 ml total) for crude exosomes (500 μl) as described above, while cells were harvested for cell lysates. A. Crude exosomes (40 μl) were analyzed by Western blotting using anti-TSG101 antibody. GFP and CD81.GFP were visualized at a wavelength of 488 nm. B & C. The remaining 460 μl crude exosomes from gagi-transfected cells were loaded on top of 6%-18% OptiPrep gradient centrifugation followed by fractionation as described above. Aliquot of each fraction was used for AChE activity assay (24 μl) (B). The remaining sample of each fraction was diluted in 4 ml PBS and spun at 100,000 g, 70 min. The pellets were lysed in the RIPA buffer followed by Western blotting using indicated antibodies (C). p24.GFP were visualized by 488 nm detection (A & C). The AChE activity was mean ± SD of duplicate samples. The data were representative of three independent experiments. (TIF) [file pone.0124436.s005.tif]
